# Clinical observation of thrombolytic effect of alteplase combined with butylphthalide in patients with acute anterior circulation cerebral infarction

**DOI:** 10.12669/pjms.37.4.3986

**Published:** 2021

**Authors:** Fan-xing Qi, Ying Hu, Sen Wang

**Affiliations:** 1 Fan-xing Qi, Department of Neurology, Baoding First Central Hospital, Baoding 071000, Hebei, China; 2 Ying Hu, Department of Cardiology, Baoding First Central Hospital, Baoding 071000, Hebei, China; 3 Sen Wang, Department of Neurology, Baoding First Central Hospital, Baoding 071000, Hebei, China

**Keywords:** Alteplase, Butylphthalide, Acute cerebral infarction, Therapeutic effect

## Abstract

**Objective:**

This study aims to evaluate the clinical effect of alteplase combined with butylphthalide in treating patients with acute anterior circulation cerebral infarction.

**Methods:**

Retrospective study methods were used. Eighty patient cases with acute anterior circulation cerebral infarction treated in Baoding First Central Hospital, China from January 2018 to December 2020 were randomly and averagely divided into two groups. Patients in the two groups were given symptomatic treatment. Patients in the experimental group were treated with alteplase combined with butylphthalide for thrombolytic therapy, whereas patients in the control group were treated with urokinase thrombolytic therapy. The NIHSS score, effective rates and neurological function recovery were analysed one day, seven days and 30 days after treatment were analyzed, respectively. So as the incidence of adverse reactions within seven days after drug adminutesistration.

**Results:**

The NIHSS scores of the two groups were significantly lower than those before treatment on one day, seven days and 30 days after treatment (experimental group, *p*=0.00; control group, *p*=0.02). The experimental group was more significantly lower than the control group (*p*=0.00). The effective rate of the experimental group was significantly higher than that of the control group (*p*=0.03), and the recovery rate after treatment was significantly higher than that of the control group (*p*=0.04). Within one week after treatment, the complication rate was 15% in the experimental group and 20% in the control group but was not significantly different (*p*=0.56).

**Conclusion:**

Alteplase combined with butylphthalide is effective and safe in the treatment of acute anterior circulation cerebral infarction without obvious complications.

## INTRODUCTION

The changes in people’s lifestyle and eating habits have contributed to the increasing incidence of acute ischaemic cerebrovascular disease. This disease has become the primary factor affecting human health and life.^1^ Eighty per cent of patients with ischaemic cerebrovascular diseases have anterior circulatory cerebral infarction.^2^ Two important therapeutic measures are currently taken for acute ischaemic cerebrovascular diseases: endovascular therapy and thrombolytic therapy.^3^ They are considered safe and effective for treating patients with acute infarction by Deguchi et al.^4^ However, the extensive application of endovascular therapy in clinical practice is limited by the need for certain instruments and surgical techniques. Alteplase is a thrombolytic drug commonly used in clinical practice, which can effectively rescue brain tissue in ischaemic penumbra for 4.5 hours.^5^ It is believed that ischaemic stroke is a complex disease involving multiple mechanisms. The multi-target therapeutic regimen may be more effective than the single-target drug therapeutic regimen.^6^ Numerous clinical studies have shown that butylphthalide’s unique effect improves patients’ symptoms with acute anterior circulation cerebral infarction and benefits their prognosis. Butylphthalide’s mechanism in ischaemic cerebrovascular disease treatment is multi-targeted and may target different pathophysiological processes, such as antioxidant, anti-inflammatory, anti-apoptotic, anti-thrombotic and mitochondrial protective processes.^7^ In this study, alteplase combined with butylphthalide was used in the treatment of acute anterior circulation cerebral infarction. The specific conditions were reported as follows.

## METHODS

### Ethical approval

The study was approved by the Institutional Ethics Committee of Baoding First Central Hospital at December 8^th^, 2020, and written informed consent was obtained from all participants.All the patients were under the age of 40.

### Inclusion criteria:

1. Onset time less than six hours.
2. Patients who meet the diagnostic criteria for acute cerebral infarction^8^ and have neurological symptoms and signs greater than one hour.
3. Moderate stroke and above (NIHSS score ≥ 5 points).^9^
4. No intracranial hemorrhage.
5. Patients or family members who agree to the research protocol and sign the consent form, and can cooperate with the research work.

### Exclusion criteria:

1. Patients with previous history of intracranial haemorrhage or subarachnoid haemorrhage.
2. Recent history of haemorrhage diseases (within three months).
3. Patients with severe hypertension (Systolic blood pressure in excess of 180mmHg, or diastolic blood pressure in excess of 110mmHg).
4. Total platelet count less than 100 × 109/L.
5. Patients with severe abnormal coagulation function (Accompanied by skin lesions, submucosal bleeding, gingival bleeding, gastrointestinal or other viscera bleeding).
6. Patients with severe heart and liver diseases that cannot be controlled satisfactorily.

Retrospective analysis was conducted. Eighty patients with acute anterior circulation cerebral infarction admitted to our hospital from January 2018 to December 2020 were randomly divided into two groups, with 40 cases in each group. The experimental group enrolled 24 males and 16 females, aged from 56 to 74 years, with an average age of 64.85 ± 6.67 years old. The control group enrolled 21 males and 19 females, aged from 53 to 72 years, with an average age of 63.55 ± 7.06 years old. There was no significant difference in general information between the two groups (Table-I).

**Table-I:** Comparative analysis of general information between experimental group and control group (*X̅* ±S) n = 40.

Indicators	Experimental group	Control group	t/χ^2^	P
Age	64.85±6.67	63.55±7.06	0.85	0.40
Male (%)	24(52.5%)	21(50%)	0.46	050
Infarct site				
Internal carotid artery (%)	8(20%)	9(22.5%)	0.07	0.78
Middle cerebral artery M1 (%)	22(55%)	23(57.5%)	0.05	0.82
Middle cerebral artery M2 (%)	10(25%)	8(20%)	0.28	0.59
NIHSS score	19.80±6.78	18.79±7.37	0.64	0.52

P>0.05

### Treatment methods

Patients in the experimental group were treated with alteplase combined with butylphthalide for thrombolytic therapy: alteplase 0.9 mg/kg, 10% of the total dose was intravenously injected within two minutes, and the remaining 90% dose was given intravenous drip with infusion pump within one hour, with the maximum dose not exceeding 100 mg. Butylphthalide 25 mg was given intravenously for more than 50 minutes, bid, with an interval of no less than six hours for two consecutive weeks. Patients in the control group were treated with urokinase thrombolytic therapy: urokinase one million U + 100 ml normal saline was intravenously injected for 30 minutes. In addition to the above thrombolytic therapy, both groups were given symptomatic treatment, such as decreasing intracranial pressure, alleviating cerebral oedema, eliminutesating oxygen free radicals, correcting water and electrolyte disturbances, providing nutritional support and nourishing brain cells.

### Observation indicators:

### 1) Efficacy observation

The therapeutic effects of the two patient groups were compared, and the changes in the NIHSS scores of the two groups before treatment and one day, seven days and 30 days after treatment were compared and analysed. *Efficiency analysis*: A comprehensive analysis was conducted on patients 30 days after treatment based on their NIHSS scores and symptom improvement.^10^ *Remarkable effect*: Symptoms and physical signs disappeared and NIHSS scores improved by more than 90%; Effect: Symptoms and physical signs improved significantly and NIHSS scores improved from 30% to 90%; No effect: No significant improvement in symptoms and physical signs and improvement of NIHSS score of less than 30%. Its effective rate is equal to the sum of the remarkable effect and the effect.

### 2) Neurological function recovery

The modified RANKIN Scale (mRs)^11^ was used to compare the neurological function recovery of the two groups 30 days after thrombolysis. An mRS score <2 was considered a good recovery, and a score of ≥2 as a poor recovery.

### 3) Adverse reactions

The incidence of adverse reactions within seven days after the medication was compared between the two groups.

### Statistical analysis

All data were statistically analysed with SPSS 20.0 software, and the measurement data were expressed as (*X̅* s). Two independent sample *t*-tests were used for inter-group data analysis. A repeated-measures analysis of variance was used for intra-group data analysis, and a χ^2^ was used for rate comparison. *P* <0.05 indicates a statistically significant difference.

## RESULTS

The changes in NIHSS scores of the two groups before and after treatment are shown in Table-II. According to the results, the patients’ scores in the two groups were significantly increased before treatment but were not significantly different. The scores decreased significantly one day, seven days and 30 days after treatment compared with those before treatment (the experimental group, *p*=0.00; the control group, *p*=0.02). The experimental group experienced a more significant reduction than the control group (*p* =0.00).The two groups’ effective rate analysis indicated that the experimental group’s effective rate was 80%, and that of the control group was 57.5%. The experimental group’s effective rate was significantly higher than that of the control group, with a statistically significant difference (*p*=0.03, see Table-III).

**Table-II:** Comparative analysis of NIHSS scores in the two groups before and after treatment (*X̅* ±S) n=40

Group	Before treatment*	1d after treatmentΔ	7d after treatmentΔ	30d after treatmentΔ	F	P
Experimental groupΔ	19.80±6.78	8.27±1.72	7.03±1.42	5.06±1.05	11.55	0.00
Control groupΔ	18.79±7.37	11.03±2.53	9.31±1.65	7.41±1.18	6.30	0.02
t	0.64	5.72	6.62	9.41		
p	0.52	0.00	0.00	0.00		

* p>0.05, Δp<0.05

**Table-III:** Effective rate analysis of two the groups (*X̅* ±S) n = 40.

Group	Remarkable effect	Effect	No effect	Efficacy rate
Experimental group	13	19	8	32(80%)
Control group	10	13	17	23(57.5%)
χ^2^				4.71
P				0.03

p<0.05

Approximately 67.5% of patients in the experimental group recovered well after treatment, compared with approximately 45% in the control group. The difference between the two groups was statistically significant (*p*=0.04, Table-IV).The incidence of complications was 15% in the experimental group and 20% in the control group within one week after treatment. Although the experimental group’s incidence was lower than that of the control group, it was not significantly different (*p*=0.56, Table-V).

**Table-IV:** Comparative analysis of neurological function recovery of the two groups before and after treatment (*X̅* ±S) n = 40.

Indicators	Desirable	Undesirable	Desirable rate
Experimental group	27	13	67.5%
Control group	18	22	45%
χ^2^			4.11
p			0.04

p<0.05

**Table-V:** Comparative analysis of the incidence of complications between the two groups (*X̅* ±S) n = 40

Group	Intracranial hemorrhage	Bleeding from other organs	Allergy	Reinfarction	Incidence rate
Experimental group	1	2	3	0	15%
Control group	2	4	1	1	20%
χ^2^					0.35
p					0.56

p>0.05

## DISCUSSION

Acute cerebral infarction is caused by abnormal vascular structure, blood composition or hemodynamics, which leads to abnormal stenosis or obstruction of cerebral artery, and rapid cerebral ischemia in a short period of time.^12^ Patients may manifest with hemiplegia, language disorders, blurred vision, involuntary movement, disturbance of consciousness. The disease will progress rapidly in a short period, leading to poor prognosis, disability and even death.^13^

In 1995, the first standard clinical trial of intravenous thrombolytic therapy was completed at the Institute of Neurological Diseases and Stroke of the National Institutes of Health. Intravenous thrombolytic therapy has gradually become the standard treatment for ischaemic stroke and is the highest recommended level in various clinical guidelines.^14^ Patients should see a doctor in time after onset and strive for the opportunity of intravenous thrombolysis within the time window to achieve a better prognosis and quality of life.^15^

Urokinase and alteplase (rt-PA) are commonly used thrombolytic drugs in the clinic. rt-PA is an endogenous enzyme with high specificity for fibrin. rt-PA activates fibrin in the blood by binding to the fibrin in a thrombus and degrading the fibrin. This local thrombolytic effect can be further supported because rt-PA is mainly activated locally in the thrombus.^16^ According to Al-Khaled et al.^17^, positive effects may also be produced in the recovery of neurological function in patients with acute ischaemic cerebrovascular disease by intravenous injection of rt-PA. In addition, Maier et al.^18^ reported that rt-PA treatment can improve eligible patients’ functional prognoses., Nevertheless, further research is needed to confirm and determinutese the patient conditions most likely to benefit from rt-PA. Among them, the blood glucose level is the primary factor that affects the thrombolytic effect. The higher the blood glucose level is before adminutesistering thrombolytic drugs such as rt-PA, the worse the therapeutic effect is. However, controlling blood glucose within a certain range is conducive to improving the therapeutic effect of thrombolytic drugs.^19^

Because of ischaemic cerebrovascular diseases’ multi-mechanism pathogenesis, including local cerebral ischaemia and hypoxia, brain cells show an electrochemical, cascade-like reaction. This reaction is coupled to signalling pathways in damaged brain cells, thereby leading to functional impairment of the central nervous system.^20^ For this reason, multi-targeted drugs are of great significance in the treatment of this disease. Butylphthalide has become an important auxiliary means for treating ischaemic cerebrovascular diseases and has been proved to improve severe cognitive dysfunction syndrome caused by insufficient brain tissue perfusion.^21^ Butylphthalide may have the following mechanism in ischaemic cerebrovascular disease treatment: reducing the nervous system damage and preventing the blood-brain barrier destruction by inhibiting M1 microglia/macrophages from transforminutesg into the M2 phenotype.^22^ Butylphthalide sequential therapy is a highly safe therapy that can effectively increase the 3-MST plasma level and reduce the A β42 plasma level, conducive to improving patients’ living ability and nerve function and facilitating their activities of daily living.^23^

It was suggested by our research that although the NIHSS scores of the two groups improved after treatment compared with those before treatment (*p*<0.05), the improvement was more obvious in patients who received alteplase combined with butylphthalide (*p*=0.00). Approximately 67.5% of patients in the experimental group recovered well regarding their neurological function after treatment, and approximately 45% in the control group (*p*=0.04) recovered. However, the difference in the incidence of complications between the two groups within one week after treatment was not significant (*p*=0.56).

### Limitations of this study

Nevertheless, this research has some shortcominutesgs. Fewer cases were included in the research, and the follow-up time was short. In general, only patients within six hours of onset were analysed. There was no further refinement of the therapeutic effect and prognosis of alteplase combined with butylphthalide in patients with different time windows. No further refinements have been made regarding the therapeutic effects and prognosis of patients with alteplase combined with butylphthalide in different time windows. In response to this, various countermeasures are being taken: cases are being actively accumulated, patient treatment data with different time windows are further improved, and follow-up times are increased. These countermeasures aim to elaborate on the shortcominutesgs and long-term effects of this therapeutic regimen in more detail.

## CONCLUSION

Alteplase combined with butylphthalide is a safe and effective treatment for patients with acute anterior circulation cerebral infarction, without obvious complications. They are worthy of clinical application.

### Authors’ Contributions:

**FQ** **and** **YH** designed this study and prepared this manuscript, and are responsible and accountable for the accuracy or integrity of the work.

**YH** collected and analyzed clinical data.

**SW** significantly revised this manuscript.

## References

[1] Seyman E, Shaim H, Shenhar-Tsarfaty S, Jonash-Kimchi T, Bornstein NM, Hallevi H (2016). The collateral circulation determinuteses cortical infarct volume in anterior circulation ischemic stroke. BMC Neurol.

[2] Burnell AL, Ranta A, Wu T, Fink J, McGuinness B, Caldwell J (2018). Endovascular clot retrieval for acute ischaemic stroke in New Zealand. N Z Med J.

[3] Yi TY, Chen WH, Wu YM, Zhang MF, Lin DL, Lin XH (2018). Adjuvant intra-arterial rt-PA injection at the initially deployed solitaire stent enhances the efficacy of mechanical thrombectomy in acute ischemic stroke. J Neurol Sci.

[4] Deguchi I, Mizuno S, Kohyama S, Tanahashi N, Takao M (2018). Drip-and-Ship Thrombolytic Therapy for Acute Ischemic Stroke. J Stroke Cerebrovasc Dis.

[5] Wardlaw JM, Murray V, Berge E, del Zoppo GJ (2014). Thrombolysis for acute ischaemic stroke. Cochrane Database Syst Rev.

[6] Chen HS, Qi SH, Shen JG (2017). One-Compound-Multi-Target:Combination Prospect of Natural Compounds with Thrombolytic Therapy in Acute Ischemic Stroke. Curr Neuropharmacol.

[7] Wang S, Ma F, Huang L, Zhang Y, Peng Y, Xing C (2018). Dl-3-n-Butylphthalide (NBP):A Promising Therapeutic Agent for Ischemic Stroke. CNS Neurol Disord Drug Targets.

[8] Takahashi M, Hashimoto M, Uehara M (2018). Preparation of a Small Acute-phase Cerebral Infarction Phantom for Diffusion-weighted Imaging. Nihon Hoshasen Gijutsu Gakkai Zasshi.

[9] Hwang J, Lee MJ, Chung JW, Bang OY, Kim GM, Chung CS (2018). NIHSS sub-item scores predict collateral flow in acute middle cerebral artery infarction. Interv Neuroradiol.

[10] Schurig J, Haeusler KG, Grittner U, Nolte CH, Fiebach JB, Audebert HJ (2019). Frequency of Hemorrhage on Follow Up Imaging in Stroke Patients Treated With rt-PA Depending on Clinical Course. Front Neurol.

[11] Jansen IGH, Mulder MJHL, Goldhoorn RB (2018). MR CLEAN Registry investigators. Endovascular treatment for acute ischaemic stroke in routine clinical practice:prospective, observational cohort study (MR CLEAN Registry). BMJ.

[12] Sveinsson ÓÁ, Kjartansson Ó, Valdimarsson EM (2014). Heilablóđþurrđ/-drep - greining og međferđ[Cerebral ischemia/infarction - diagnosis and treatment]. Laeknabladid.

[13] Kufner A, Galinovic I, Ambrosi V, Nolte CH, Endres M, Fiebach JB (2015). Hyperintense Vessels on FLAIR:Hemodynamic Correlates and Response to Thrombolysis. Am J Neuroradiol.

[14] Haverkamp C, Ganslandt T, Horki P, Boeker M, Dorfler A, Schwab S (2018). Regional Differences in Thrombectomy Rates :Secondary use of Billing Codes in the MIRACUM (Medical Informatics for Research and Care in University Medicine) Consortium. Clin Neuroradiol.

[15] Hohenhaus M, Schmidt WU, Brunecker P, Xu C, Hotter B, Rozanski M (2012). FLAIR vascular hyperintensities in acute ICA and MCA infarction:a marker for mismatch and stroke severity?. Cerebrovasc Dis.

[16] Saver JL, Goyal M, Bonafe A, Diener HC, Levy EI, Pereira VM (2015). Solitaire™with the Intention for Thrombectomy as Primary Endovascular Treatment for Acute Ischemic Stroke (SWIFT PRIME) trial:protocol for a randomized, controlled, multicenter study comparing the Solitaire revascularization device with IV tPA with IV tPA alone in acute ischemic stroke. Int J Stroke.

[17] Al-Khaled M, Brüning T, Gottwald C, Roessler F, Royl G, Eckey T (2018). Comparing outcome and recanalization results in patients with anterior circulation stroke following endovascular treatment with and without a treatment with rt-PA:A single-center study. Brain Behav.

[18] Maier IL, Behme D, Schnieder M (2017). Bridging-therapy with intravenous recombinant tissue plasminutesogen activator improves functional outcome in patients with endovascular treatment in acute stroke. J Neurol Sci.

[19] Zhang Z, Qian M, Ge Z, Zhou P, Liu J, Chen J (2019). Effects of blood glucose and glycosylated hemoglobin levels on intravenous thrombolysis in patients with acute cerebral infarction and type 2 diabetes mellitus. Pak J Med Sci.

[20] Stampfl S, Ringleb PA, Haehnel S, Rocco A, Herweh C, Hametner C (2013). Recanalization with stent-retriever devices in patients with wake-up stroke. Am J Neuroradiol.

[21] Chen XQ, Qiu K, Liu H, He Q, Bai JH, Lu W (2019). Application and prospects of butylphthalide for the treatment of neurologic diseases. Chin Med J (Engl).

[22] Zhao YJ, Nai Y, Li SY, Zheng YH (2018). Retigabine protects the blood-brain barrier by regulating tight junctions between cerebral vascular endothelial cells in cerebral ischemia-reperfusion rats. Eur Rev Med Pharmacol Sci.

[23] Wo X, Han J, Wang J, Wang X, Liu X, Wang Z (2020). Sequential butylphthalide therapy combined with dual antiplatelet therapy in the treatment of acute cerebral infarction. Pak J Med Sci.

